# Initial retrieval shields against retrieval-induced forgetting

**DOI:** 10.3389/fpsyg.2015.00657

**Published:** 2015-05-21

**Authors:** Mihály Racsmány, Attila Keresztes

**Affiliations:** ^1^Department of Cognitive Science, Budapest University of Technology and Economics, Budapest, Hungary; ^2^Research Group on Frontostriatal Disorders, Hungarian Academy of Sciences, Budapest, Hungary; ^3^Center for Lifespan Psychology, Max Planck Institute for Human Development, Berlin, Germany

**Keywords:** retrieval-induced forgetting, retrieval-enhanced learning, inhibition, context reinstatement, episodic memory, context effects

## Abstract

Testing, as a form of retrieval, can enhance learning but it can also induce forgetting of related memories, a phenomenon known as retrieval-induced forgetting (RIF). In four experiments we explored whether selective retrieval and selective restudy of target memories induce forgetting of related memories with or without initial retrieval of the entire learning set. In Experiment 1, subjects studied category-exemplar associations, some of which were then either restudied or retrieved. RIF occurred on a delayed final test only when memories were retrieved and not when they were restudied. In Experiment 2, following the study phase of category-exemplar associations, subjects attempted to recall all category-exemplar associations, then they selectively retrieved or restudied some of the exemplars. We found that, despite the huge impact on practiced items, selective retrieval/restudy caused no decrease in final recall of related items. In Experiment 3, we replicated the main result of Experiment 2 by manipulating initial retrieval as a within-subject variable. In Experiment 4 we replicated the main results of the previous experiments with non-practiced (Nrp) baseline items. These findings suggest that initial retrieval of the learning set shields against the forgetting effect of later selective retrieval. Together, our results support the context shift theory of RIF.

## Introduction

The act of retrieval facilitates later access to retrieved memories. Typically, in comparison with repeated study (restudy), repeated retrieval of memories improves long-term retention, whereas it produces equal or often lower recall performance following a short-term delay (Carrier and Pashler, 1992; Wheeler et al., 2003; Roediger and Karpicke, 2006a,b; Karpicke and Roediger, 2008; Toppino and Cohen, 2009; Keresztes et al., 2013). However, the long-term benefits of retrieval often come with a cost: retrieval-induced forgetting (RIF; Anderson et al., 1994); when retrieval is selective, non-retrieved, but related memories become less accessible.

It has been shown that both selective retrieval and selective restudy of a learning set increase the recall probability of retrieved/restudied memories; however, only selective retrieval induces forgetting of related information from the same set (Ciranni and Shimamura, 1999; Anderson et al., 2000; Bäuml, 2002; Bäuml and Aslan, 2004; Staudigl et al., 2010; but see Verde, 2009). RIF is a robust experimental phenomenon at short delays, and recent findings suggest that it is present also after longer delays (Racsmány et al., 2010; Abel and Bäuml, 2012; Storm et al., 2012; but see MacLeod and Macrae, 2001).

Importantly, this pattern of findings is a potential problem for any educational program using frequent selective retrieval—i.e., testing—of large sets of information as a learning method. In brief, these findings highlight that retrieval has a robust long-term advantage over repeated study of information at the expense of forgetting related, but not retrieved, information. Identifying any factor that could protect these memories from being forgotten, therefore, is key to creating effective learning programs.

In the following sections, we outline the retrieval practice paradigm (Anderson et al., 1994), that is most commonly used to investigate RIF, and then briefly overview three families of theories on associative retrieval processes that can explain RIF. Finally, based on the assumptions of one family of theories, we suggest one critical factor that could shield against the adverse effects of RIF: an initial—non-selective—retrieval of the entire learning set.

In the retrieval practice paradigm (Anderson et al., 1994), participants study category–member pairs (e.g., animal–tiger, furniture–couch, animal–chicken, etc.); then, in a selective retrieval practice phase, they repeatedly retrieve half of the members from half of the categories (e.g., animal–t…?). Typically, final recall administered after a delay reveals that repeated selective retrieval leads to forgetting of related material (e.g., “animal–c…?”) compared to unpracticed baseline categories (e.g., furniture–c…?)—this effect is referred to as RIF.

The most influential family of theories—the inhibitory control based accounts—posit that when participants practice retrieval of half of the members from a given category, the other half would compete for retrieval (Anderson et al., 1994, 2000; Anderson and McCulloch, 1999; Anderson and Bell, 2001; Bäuml and Hartinger, 2002; Storm et al., 2006; Storm and Nestojko, 2010). This competition is then resolved by executive control guided active inhibition, which renders the memories of competitors less accessible for later recall (Anderson, 2005; Anderson and Levy, 2007).

Interference based accounts—the second family of theories—explain RIF without inhibition (Camp et al., 2007, 2009; Jakab and Raaijmakers, 2009). These models assume that strengthening some category-member associations is enough to lead to interference at any later attempt to retrieve competitors. Here, it is this interference at final recall that leads to RIF. The most influential of these models, the search of associative memory (SAM) model (Raaijmakers and Shiffrin, 1981) assumes that retrieval occurs in two steps. First—in the sampling phase—cues are assembled into a short-term store for activated memory sets, and items are sampled into these sets based on the relative strength of their associations to the given cue. In a second step—the recovery phase—sampled items are retrieved based on the absolute strength of their associations to the given cue. It is only a successful recovery that leads to conscious retrieval of a memory item. Using these terms, interference based accounts assume that RIF is the consequence of a sampling failure, i.e., a bias in relative associative strengths, whereas inhibitory models assume that RIF occurs due to recovery failure, i.e., due to a decreased item strength.

The third family of theories pinpoint episodic or context-based retrieval as the source of RIF, suggesting that any kind of retrieval creates and reshapes highly contextualized episodic memory representations (Racsmány and Conway, 2006; Conway, 2009; Racsmány et al., 2012; Jonker et al., 2013; Karpicke et al., 2014; see Sahakyan and Hendricks, 2012, for a similar account of directed forgetting). Episodic memory sets contain context, cue, and item features (Racsmány and Conway, 2006; Conway, 2009). The most influential of these theories emphasizes the role of context shift between studying a memory set and retrieval of parts of this set (Jonker et al., 2013; for a similar account of directed forgetting, see Sahakyan and Kelley, 2002). According to the context shift theory, the mental context of the study phase is changed in the following retrieval phase due to processes activated by retrieval of parts of the set. This context then remains the same throughout the rest of these experiments—RIF is found because the mental context of the final recall is biased to mimic retrieval pattern of the previous selective retrieval and not that of the initial study phase.

Importantly for our current research question, the context shift theory leads to the prediction that an initial retrieval attempt of the entire learning set can eliminate the adverse effect of later selective retrieval. This is because an initial retrieval can already establish the episodic context for the rest of the experiment (see Jonker et al., 2013; Karpicke et al., 2014). This way, final recall will bias the retrieval process to mimic the pattern of the initial retrieval and grant access to items not selectively practiced as well.

Retrieval is so central to the wide range of the above discussed theories that retrieval-specificity—the concept that retrieval is necessary to produce RIF—has become a descriptive feature of RIF (Anderson and Spellman, 1995; Anderson, 2003; Storm, 2011). A crucial, and well replicable finding, is that selectively restudying category-member pairs is not enough to produce RIF, category members should be selectively retrieved to induce the effect (Blaxton and Neely, 1983; Bäuml, 1996, 1997, 2002; Ciranni and Shimamura, 1999; Anderson et al., 2000; Anderson and Bell, 2001; Shivde and Anderson, 2001; Levy and Anderson, 2008; Jonker et al., 2013;, but see Raaijmakers and Jakab, 2012). This finding is in line with the inhibitory control based accounts, because these assume that inhibition is only necessary when the retrieval process induces competition between target memories and competitors (Anderson, 2003). It is also in line with theories emphasizing the role of context-based, episodic retrieval in producing RIF, because these theories assume that it is the retrieval process that produces the shift from the study context to the context of retrieval, and creates biased contextualized episodic memory sets (Racsmány and Conway, 2006; Jonker et al., 2013). In contrast, according to the interference accounts, both selective retrieval and restudy should lead to RIF—a prediction incompatible with what is generally found.

However, Verde (2013) suggested that the latest version of the SAM–REM model (Malmberg and Shiffrin, 2005) could explain the same pattern with the additional assumption that retrieval strengthens the context-item associations, whereas restudy strengthens cue-item associations. Because only the former affects the sampling process (by modifying relative strength of associations)—the source of RIF in this model—only retrieval leads to RIF. In support of this suggestion, recent studies (Jonker and MacLeod, 2012; Raaijmakers and Jakab, 2012; Verde, 2013; Experiment 2) showed that selectively strengthening category-member associations and emphasizing context encoding without retrieval might also lead to RIF.

Given the pivotal role of retrieval in shaping episodic memory sets, it is surprising that studies using the retrieval practice paradigm have not investigated the effect of an initial retrieval phase where participants attempt to recall the entire learning set once before selective retrieval. To our knowledge, in the vast amount of experiments investigating the RIF effect, the first retrieval act that occurred in the experiments was selective retrieval, when participants aimed to access only a part of the studied elements^1^.

Besides investigating its protective role against RIF, performance in an initial retrieval phase could also provide experimenters with a direct baseline for measuring the extent of forgetting. In the retrieval practice paradigm, baseline is generally measured as the final recall performance of memory items belonging to categories not appearing during the practice phase. Because these categories and corresponding target memories appear in the initial study phase, but neither the category label, nor any member of these categories appear during the selective practice manipulation, these items seem to be a good choice for measuring baseline performance. However, this poses at least three problems in the interpretation of final recall performance. The first is baseline deflation (Anderson, 2003), coined for the phenomenon that during the course of a test session items tested later will suffer interference from items tested earlier, and the probability of successful recall during a test session decreases with the number of previously tested items. The second is cue priming: Cues for selectively retrieved categories appear during the practice phase, and this causes a bias in cue processing at final recall so that practiced items are more probably retrieved and may block access to unpracticed items. Similar cue biases do not occur for cues of categories not selectively retrieved. Third, context biases may add up to cue priming: The context of the retrieval practice phase itself creates uneven recall probabilities for retrieved and non-retrieved memories from categories retrieved during the practice phase. Again, similar context biases do not occur for cues of categories not retrieved during the practice phase of the retrieval practice paradigm (Racsmány and Conway, 2006; Jonker et al., 2013). We suggest that measuring baseline directly with an initial retrieval of the entire learning set can circumvent these issues, and facilitate interpretation of final recall data in the retrieval practice paradigm.

In this paper, we investigated the possible adverse effect of retrieval practice on a part of the studied elements when an initial retrieval accessed the entire memory set studied earlier in the experiment. Additionally, using performance of this initial retrieval, the effect of further selective retrieval on both retrieved and non-retrieved memories could be assessed to a baseline recall level of the same memories. Therefore the following experiments had two aims: first, to measure the interaction between initial testing of the entire learning set and the adverse effect of later selective retrieval practice on related unpracticed items, and second, to introduce a novel baseline measure, the initial retrieval performance, for future RIF experiments.

Based on accounts emphasizing the episodic/contextual nature of retrieval practice (Racsmány and Conway, 2006; Jonker et al., 2013; Karpicke et al., 2014), we predicted that an initial attempt to—non-selectively—retrieve the entire learning set would shield against the adverse effects of later selective retrieval, together with maintaining the positive effects of retrieval practice for retrieved memories. In contrast, interference accounts would predict no effect of an initial retrieval. Because in these accounts, RIF depends on relative cue-item or context-item association strengths, an equally distributed increase in these association strengths would not shift the effect of later selective strengthening of these associations. It is harder to derive predictions based on inhibitory control based accounts. Although strengthening all items via an initial retrieval can lead to larger competition during later selective retrieval—hence to larger RIF, the effect could also be the opposite; based on a trade-off between the need for inhibition during competitive retrieval, and the success of inhibition (Norman et al., 2007; Anderson and Levy, 2011; see experimental evidence, Keresztes and Racsmány, 2013) it can well be that strengthening items that later become competitors can render inhibitory processes ineffective—hence to no RIF. Similarly, results showing that retrieval of cue-item associations can decrease later interference generated by these associations (Szpunar et al., 2008; Halamish and Bjork, 2011) would suggest that an initial retrieval of competitors can decrease competition during later selective retrieval of related targets. Again, decreased competition would lead to decreased inhibition—hence to an attenuated RIF.

The first experiment reported here aimed to replicate previous findings of retrieval specificity of RIF. Then, using the same material and procedures, we investigated the effect of an initial retrieval of all items in the experiment on further effects of selective retrieval.

## Experiment 1

### Method

#### Participants

All four experiments were approved by the Ethical Committee of the Budapest University of Technology and Economics, and all participants gave their written informed consent.

Sixty^2^ participants were recruited for Experiment 1 at the Budapest University of Technology and Economics. Outliers were defined as data points more than three standard deviations away from the group mean. We screened data for outliers for overall recall performance and recall in all four item types (see design section). Data for one participant was identified as outlier; and excluded from further analyses. Therefore, the results section shows the data for 59 participants (26 men and 28 women), aged between 19 and 26 years (*M* = 20.36, SD = 1.47).

#### Design and Materials

We varied practice type (retest or restudy) between subjects, and item type within subjects. We used 10 categories and six words from each category, a total of 60 category-word pairs. To induce competitive retrieval supposed to be necessary to produce RIF, and to avoid moderation of the RIF effect (see Anderson, 2003), we followed strict selection criteria described in detail in Keresztes and Racsmány (2013). Briefly, we used neutral words of moderate frequency, based on the Frequency Dictionary of the Hungarian Webcorpus (Halácsy et al., 2004; Kornai et al., 2006). We used categories that were not associated to each other (either semantically or phonetically), and category members that were not associated to another member of another category.

Members of two categories were used as filler items. The remaining 48 words from the remaining eight categories were assigned to one of the four item types. Counterbalancing across all conditions was achieved by a full randomization procedure run by Presentation^®^ software (Version 14.7, www.neurobs.com) for each participant separately. Briefly, four categories were selected randomly to be practiced categories. The four others were to be unpracticed categories. Words within each category were split randomly into two groups. One half of the words (Rp+) in each practiced category was to be practiced during the practice phase, the other half (Rp–) was not. Words in the unpracticed categories were used as baseline items. One half of the words (Nrp+) in each unpracticed category served as baseline for Rp+ words, the other half (Nrp–) served as baseline for Rp– words.

#### Procedure

The experiment consisted of four phases: a study phase, a practice phase, a delay, and a final test phase. Restudy and retest conditions differed only in their practice phase.

In the study phase, participants were presented all 60 words paired with their category label. Each pair was shown once for 5000 ms in the centre of the screen with the category label on the left and the category member on the right. Participants were instructed to memorize the words with the help of the category label. Presentation of the pairs was pseudo-randomized with the constraint that two words belonging to the same category could not appear consecutively.

The practice phase consisted of three cycles, each containing a practice block with 18 trials followed by a reexposure block with 18 trials. Practice and reexposure blocks each consisted of 12 trials with Rp+ items and six trials with filler items. The first and the last two items in each block were filler items. The order of the rest of the items was pseudorandomized with the constraint that two consecutive trials never involved members of the same category.

Practice trials in the retest condition were cued recall trials. In each trial, the category label of the target word plus a two-letter stem cue for the target word appeared in the middle of the screen, and participants were instructed to complete the stem to the corresponding target. They had 6000 ms in the first cycle and 4000 ms in the second and third cycle to type the answer using a keyboard. Practice trials in the restudy condition were the same as trials in the study phase, except that restudy trials lasted 6000 ms in the first cycle and 4000 ms in the second and third cycle. Each pair was shown once in the center of the screen with the category label on the left and the category member on the right, and participants were instructed to use these trials to restudy the category label—word pairs.

Reexposure trials were the same as trials in the study phase, except that reexposure trials lasted 1000 ms. Participants were told that they would see some words again in a rapid sequence as a memory enhancer. Note that whereas practice trials were different for the retest and restudy conditions, reexposure trials were the same. Reexposure trials served as a feedback in the retest condition, and were introduced in the restudy condition as well to equal the time on study in the two conditions.

The three practice cycles (for both retest and restudy) followed each other in a repeated spaced retrieval schedule in order to enhance the effect of testing (see Karpicke and Bauernschmidt, 2011). We introduced 1, 3, and 6 min of delay filled with a two-back task, before the first, second, and third practice cycle, respectively.

After the practice phase participants performed a 5-min long two-back task, and then were introduced to the final test phase. In the 2-back task, participants saw a series of numbers, one at a time, in the middle of a computer screen, and for each trial they had to respond by pressing a button on the keyboard when the number in the current trial was the same as the one presented two trials before. In each trial, stimuli was sampled pseudorandomly from among five integers (1–5) so that the program selected the current number to be a target, i.e., the same as the number appearing to trials before, with a 25% probability. Trials were 2000 ms long (700 ms stimulus duration, 1300 ISI). Participants received a 2000 ms feedback for hits, misses, and false alarms.

The final test consisted of two blocks. In order to avoid output interference (see Anderson, 2003) Rp– items and their controls (Nrp– items) were tested in the first block, followed by Rp+ items and their controls (Nrp+ items) in the second block. Items were randomly intermixed within blocks (Camp et al., 2007). The use of different control items for Rp+ and Rp– items was necessary to circumvent baseline deflation (see Anderson, 2003). Both blocks started and ended with two filler items. Trials were the same as in the first retrieval practice block except that the category-plus-word-stem cue contained only a first-letter stem of the category member.

Randomization of trials, presentation of stimuli, response logging, and data preprocessing were performed by Presentation^®^ software (Version 14.7, www.neurobs.com).

### Results and Discussion

**FIGURE 1:**
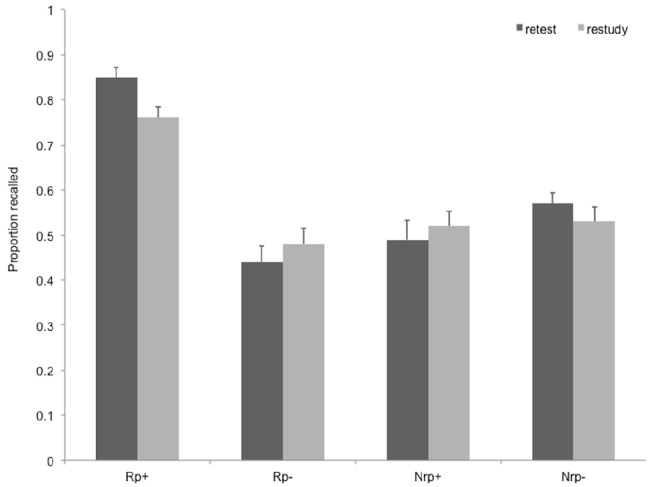
**Recall performance on the final test in Experiment 1, for the four item types in the two practice conditions.** Rp+, Practiced words from practiced categories; Rp–, unpracticed words from practiced categories; Nrp+, words from unpracticed categories used as baseline for Rp+ words; Nrp–, words from unpracticed categories used as baseline for Rp– words. Error bars indicate standard error of the mean.

Throughout the manuscript, we report effect sizes using *r* for *t*-tests and ${\text{η}}_{p}^{2}$ for *F*-tests. Recall performance at the final test for the four item types are shown in Figure 1.

#### The Effect of Practice on Final Recall

We conducted a mixed design ANOVA on recall data with item type (Rp+, Rp–, Nrp+, Nrp–) as a repeated measures variable, and practice type (retest vs. restudy) as a between subject variable. Item type had a significant main effect on final recall, *F*(3,171) = 66.40, *p* < 0.001, ${\text{η}}_{p}^{2}$ = 0.54, and there was a tendency toward an interaction of item type with practice type, *F*(3,171) = 2.53, *p* = 0.058, ${\text{η}}_{p}^{2}$ = 0.04. Retesting led to a similar overall recall as restudying, *F*(1,57) = 0.26, ns.

To detect RIF, we performed paired-samples *t*-tests for participants in the retest and the restudy condition separately, contrasting Rp– recall with Nrp– recall. The RIF effect was only significant in the retest condition, *t*(28) = –3.13, *p* = 0.004, *r* = 0.37, but no RIF was found in the restudy condition, *t*(29) = 1.43, *p* = 0.16. In brief, testing induced forgetting only when participants were retested during the practice phase, and not when they restudied the same material.

Retrieval practice led to enhancement of memory for practiced items (as compared to Nrp+ baseline items) in both conditions, *t*(28) = 5.91, *p* < 0.001, *r* = 0.60, in the restudy and *t*(29) = 9.94, *p* < 0.001, *r* = 0.70 in the retest condition.

In brief, the results of Experiment 1 replicated earlier findings: Selectively retrieving memories from a category induce forgetting of related, but non-retrieved memories from the same category, whereas selective restudy of memories does not lead to this type of forgetting. Importantly, *post hoc* power calculations on data from Experiment 1 showed that the paradigm was indeed well-powered to detect any differences between Rp– items and their Nrp– baselines (1 – β) = 0.88. It was crucial for us to have a well-powered paradigm in order to exclude Type II errors in the following experiments.

In Experiment 2 we manipulated the type of practice within subjects, and introduced an initial retrieval test immediately after the study phase to test whether an initial retrieval test able to eliminate the RIF effect. This procedure also introduced a novel baseline measure for each item type: the initial recall performance. Note that this experiment did not involve unpracticed items from unpracticed categories (NRP items) as a baseline.

## Experiment 2

### Method

#### Participants

Thirty participants were recruited at the Budapest University of Technology and Economics (15 men and 15 women), aged between 19 and 26 years (*M* = 21.9, SD = 1.88). None of them participated in Experiment 1.

#### Materials, Design, and Procedure

Materials were the same as in Experiment 1. Two differences were introduced in the design and procedure.

First, practice type (retest vs. restudy) was manipulated within subjects, so that half of the critical categories were randomly assigned to be retested and another half were assigned to be restudied. (Note that there were no categories that did not receive one kind of practice, i.e., Nrp categories were not used in this experiment.) Again, only half of the members from each category underwent practice. In the practice phase retest and restudy trials were run in separate blocks, with two blocks in each practice cycle. Within each cycle, the order of retest and restudy blocks was counterbalanced between subjects.

Second, participants were tested once for all word pair right after the study phase. Trials in this initial test phase were identical to trials in the final test phase (also identical to the test phase of Experiment 1). To our knowledge, this was the first experiment using the retrieval practice paradigm that measured baseline recall levels as the performance on the first retrieval attempt after an initial study. All other aspects of this experiment were the same as those in Experiment 1.

### Results and Discussion

#### The Effect of Practice on Final Recall

We conducted a repeated measures ANOVA on final recall data with item type (practiced vs. unpracticed) and practice type (retest vs. restudy) as repeated measures variables. Item type had a significant main effect on final recall, *F*(1,29) = 141.31, *p* < 0.001, ${\text{η}}_{p}^{2}$ = 0.83. There was no main effect of practice type, *F*(1,29) = 0.96, ns., and no interaction, *F*(1,29) = 0.004, ns. The same ANOVA on initial recall performance revealed that baseline performance did not differ in the four conditions, i.e., no main effect of item type, *F*(1,29) = 0.10, ns., practice type, *F*(1,29) = 0.25, ns., and no interaction, *F*(1,29) = 0.09, ns., emerged.

**FIGURE 2:**
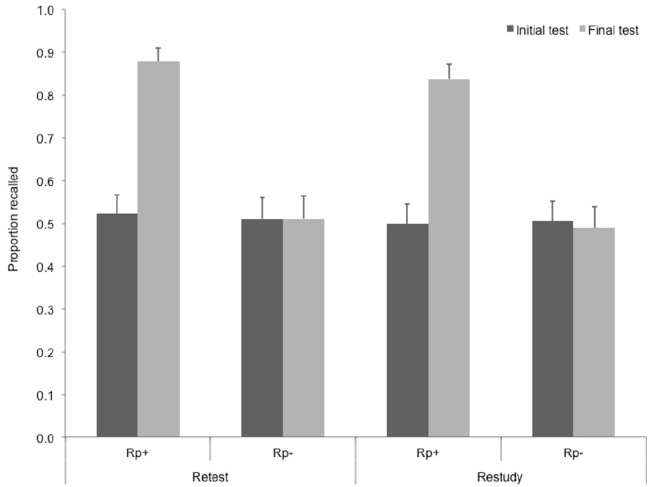
**Recall performance on the baseline test and the final test in Experiment 2, for the two item types in the two practice conditions.** Rp+, Practiced words from practiced categories; Rp–, unpracticed words from practiced categories.

To detect RIF, we performed paired-samples *t*-tests for unpracticed items vs. their own baselines, i.e., recall performance of the same items at the first retrieval attempt, in the retest and the restudy condition separately. Looking at the data in Figure 2, it is not surprising that we found no significant RIF in either the retest, *t*(29) = 0.00, ns., or the restudy condition, *t*(29) = 0.72, ns. In brief, practice, either through restudying or retesting, did not induce forgetting when items had been retrieved once after the study phase. Retrieval practice led to enhancement of memory for practiced items (as compared to their own baselines, i.e., recall performance of the same items at the first retrieval attempt) in both conditions, *t*(29) = 10.29, *p* < 0.001, *r* = 0.68, in the restudy and *t*(29) = 9.95, *p* < 0.001, *r* = 0.70, in the retest condition.

In Experiment 2, we measured initial retrieval performance. Comparing the effect of selective retrieval and selective restudy to this initial retrieval performance, we found that practicing by means of both selective retrieval and selective restudy enhanced recall of practiced memories. We also found that neither type of practice (either retrieval or restudy) impaired accessibility of memories related to the cues associated to the practiced memories. This finding is not surprising in the condition where practice involved restudy—it is consistent with finding no RIF after selective restudy in Experiment 1, as well is many other experiments (Blaxton and Neely, 1983; Bäuml, 1996, 1997, 2002; Ciranni and Shimamura, 1999; Anderson et al., 2000; Anderson and Bell, 2001; Shivde and Anderson, 2001; Levy and Anderson, 2008). However, based on the predictions of inhibitory and interference explanations of RIF, the lack of RIF is indeed surprising in the condition where practice involved retrieval. In contrast, these results are in line with contextual accounts of RIF. These accounts predict that an initial retrieval of the entire learning set after the study phase will already have participants change their mental context and change contextual memory representation of studied items and later selective retrieval practice will cause no further change in this mental context and contextual memory representation (Racsmány and Conway, 2006; Jonker et al., 2013).

Although—as shown in Experiment 1—our paradigm was well-powered to detect a RIF effect if it existed, Experiment 2 did not allow directly testing the effect of initial testing, because it did not include a condition without initial testing. The goal of Experiment 3 was to allow for directly testing the impact of an initial test on the forgetting effect induced by later selective practice.

## Experiment 3

### Method

To analyze the effect of initial retrieval test in a single experiment, participants practiced word pairs during the practice phase through retrieval practice, and we varied whether categories received an initial test or not within subjects.

#### Participants

Thirty participants were recruited at the Budapest University of Technology and Economics. One participant’s data were excluded from the analyses, because of a failure to type in the answers during the baseline test, therefore the final sample consisted of 29 individuals (16 men and 13 women), aged between 19 and 28 years (*M* = 22.68, SD = 2.59). None of them participated in previous experiments.

#### Materials, Design, and Procedure

Materials were the same as in Experiments 1 and 2. Two changes were introduced to the design and procedure of Experiment 2. First, initial test was administered only for half of the categories, and second, participants practiced by only retrieval and not by restudy for half of the words in all critical categories. Therefore this experiment did not involve a restudy condition, and item type (practiced vs. unpracticed) and initial test (administered vs. not administered) was varied within subjects. As in Experiment 2, each practice cycle contained two blocks. One block included trials with items that had received an initial test and the other block included trials with items that did not. Within each cycle, the order of blocks was counterbalanced between subjects. All other aspects of this experiment were the same as those in Experiment 2.

### Results and Discussion

#### The Effect of Practice on Final Recall

**FIGURE 3:**
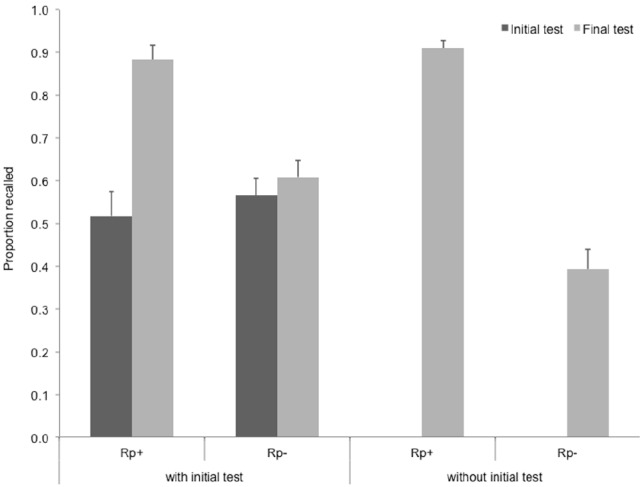
**Recall performance on the final test in Experiment 3, for the two item types in the two initial test conditions.** Rp+ items with/without initial test: practiced words from categories that were/were not tested during an initial test, Rp– with/without initial test: unpracticed words from categories that were/were not tested during an initial test. Error bars indicate standard error of the mean.

Recall performance at the final test for the four item types is shown in Figure 3, together with the initial test performance for items with initial test. A repeated measures ANOVA on final recall data with item type (practiced vs. unpracticed) and initial test (administered vs. not administered) as repeated measures variables. Item type had a significant main effect on final recall, *F*(1,28) = 203.30, *p* < 0.001, ${\text{η}}_{p}^{2}$ = 0.88, as well as the initial test, *F*(1,28) = 17.05, *p* < 0.001, ${\text{η}}_{p}^{2}$ = 0.38. Importantly, initial test interacted significantly with item type, *F*(1,28) = 31.96, *p* < 0.001, ${\text{η}}_{p}^{2}$ = 0.53. These effects occurred with initial performance not being different for practiced and unpracticed items, *t*(28) = 1.11, *p* = 0.12.

To assess RIF, we performed paired-samples *t*-tests contrasting unpracticed items and their available baselines. Note that in this experiment, we used initial test performance as a baseline—there were no categories that did not receive practice, i.e., Nrp categories were not used in this experiment. Therefore, final recall of Rp– items was contrasted with initial test performance of for Rp–, both for categories that received an initial test and for categories that did not. We found a significant RIF for Rp– items that did not receive an initial test, *t*(28) = 4.23, *p* < 0.001, *r* = 0.41, but no RIF for items that received an initial test, *t*(28) = 1.61, ns. Retrieval practice led to enhancement of memory for practiced items irrespective of whether initial test occurred or not, *t*(28) = 9.04, *p* < 0.001 for items without an initial test, *r* = 0.72, and *t*(28) = 8.46, *p* < 0.001 for items with an initial test, *r* = 0.64.

Using a within-subject design, Experiment 3 showed that initial testing can eliminate RIF due to later selective practice. However, one might argue that this experiment did not allow for calculating a classical RIF score ([Nrp–] – [Rp–]), as it did not include Nrp items. The goal of experiment 4 was to remedy this issue. This was important because the higher recall rate of Rp– items with initial test compared to recall rate of Rp– items without an initial test in Experiment 3 might have been the result of a higher rate of decay for initially non-tested information. The inclusion of unpracticed baseline items (Nrp) in Experiment 4 allowed us to test this alternative explanation.

## Experiment 4

### Method

This experiment was an extension of Experiment 1 with all items in the experiment receiving an initial test. This experiment allowed us to compare the effect of retrieval practice on the recall of different item types to a standard Nrp performance, i.e., items from categories with no selective retrieval practice, and also to an initial retrieval test performance measured for each item type.

#### Participants

Twenty-nine (13 men and 16 women), aged between 19 and 26 years (*M* = 21.66, SD = 2.16), participants were recruited at the Budapest University of Technology and Economics. None of them participated in previous experiments.

#### Materials, Design, and Procedure

Materials, design, and procedure were the same as in Experiment 1, with two major changes: First, an initial test was administered for all items. Second, participants practiced by only retrieval and not by restudy for half of the words from half the categories. The initial test phase was inserted immediately after the study phase. Trials in this initial test phase were identical to those in the same phase of Experiment 2.

### Results and Discussion

#### The Effect of Practice on Final Recall

**FIGURE 4:**
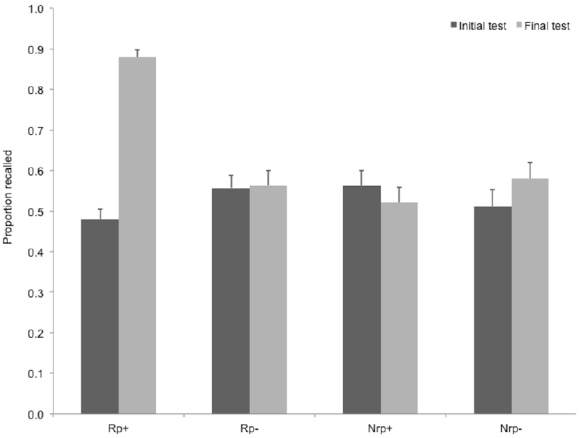
**Recall performance on the initial test and the final test in Experiment 4, for the four item types.** Rp+, Practiced words from practiced categories; Rp–, unpracticed words from practiced categories; Nrp+, words from unpracticed categories used as baseline for Rp+ words; Nrp–, words from unpracticed categories used as baseline for Rp– words. Error bars indicate standard error of the mean.

Recall performance at the final test as well as initial test performance for the four item types are shown in Figure 4. A repeated measures ANOVA on final recall data revealed a significant main effect of item type, *F*(3,84) = 36.87, *p* < 0.001, ${\text{η}}_{p}^{2}$ = 0.57. Importantly, in this experiment, we could assess RIF either by contrasting Rp– recall performance to Nrp– performance, i.e., items from categories with no selective retrieval practice, and also by contrasting initial test performance to final recall performance of the same Rp– items. Paired-samples *t*-tests showed that neither comparison yielded a significant RIF effect, *t*(28) = 0.43, ns., for the contrast with Nrp– items, and *t*(28) = 0.24, ns., for the contrast with initial Rp– recall performance. Similarly, retrieval practice led to enhancement of memory for practiced items both based on the contrast between Rp+ recall and Nrp+ recall, *t*(28) = 11.91, *r* = 0.74, and based on the contrast between Rp+ recall and initial Rp+ recall performance, *t*(28) = 22.53, *r* = 0.86.

#### Comparison of Retrieval-Induced Forgetting Across Experiments 1 and 4

**FIGURE 5:**
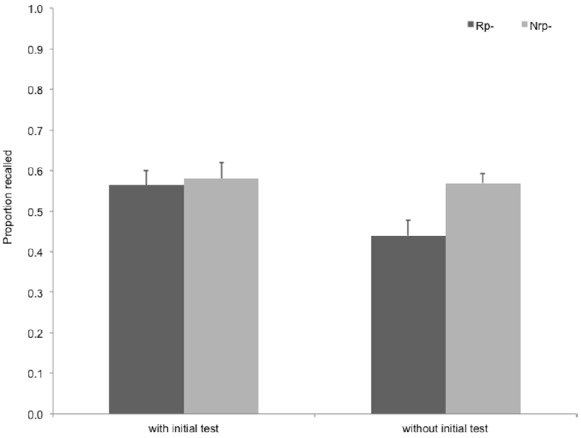
**Comparison of RIF as a function of whether an initial test took place or not.** Data without initial test was collected in Experiment 1, data with initial test (testing all items shown in the study phase once, after the study phase, before the retrieval practice phase) was collected in Experiment 4. Rp–, unpracticed words from practiced categories; Nrp–, words from unpracticed categories used as baseline for Rp– words. Error bars indicate standard error of the mean.

Collapsing data across Experiments 1 and 4 also allowed us to compare classical RIF scores ([Nrp–] – [Rp–]) across two critical conditions—one with an initial test of all Nrp– and Rp– items, and one without it. Although this analysis was *post hoc*, it could provide converging evidence for the effect of initial test on RIF. To compare RIF scores across procedures with (Experiment 4) and without (Experiment 1) an initial test, we performed a mixed design ANOVA on recall data of Rp– and Nrp– items collapsing data from the two experiments (see Figure 5). This analysis revealed a tendency for an interaction of item type (Rp– vs. Nrp–) and initial test (with vs. without initial test), *F*(1,56) = 3.842, *p* = 0.055, ${\text{η}}_{p}^{2}$ = 0.064, indicating that initial testing of all items studied in the experiments reduced the RIF effect.

Importantly, we found no difference between the final recall of Nrp items with and without initial recall, *t*(56) = 0.24, ns., showing that the initial test itself did not change the studied items’ forgetting rate. This also suggests that the different recall rates of Rp– items with and without initial test cannot be explained by faster forgetting without initial test.

## Discussion

In four experiments we investigated the interactive effect of initial retrieval of the entire learning set and later selective practice of a part of studied items on final recall performance. We found that selectively practice—either by retrieval or by restudy—increased the recall probability of the practiced items on a final recall. However, only selective retrieval practice decreased final recall of the unpracticed members from the practiced categories in comparison with exemplars of unpracticed categories (Experiment 1). An initial retrieval of the learning set shielded against the adverse effect of retrieval practice; RIF was absent either when measured to baseline performance on the initial retrieval (Experiments 2 and 3), or to members of unpracticed categories (Experiment 4).

These results can be explained by assuming that selective retrieval, by shifting the context of the study phase to the context of retrieval practice phase, leads to RIF by generating a compound contextual episodic memory representation with a restricted and biased search set (Karpicke et al., 2014). In such contextualized memory sets, cue-item associations are biased toward increased recall probabilities for retrieved items from practiced categories and decreased recall probabilities for non-retrieved items from practiced categories (Racsmány and Conway, 2006; Jonker et al., 2013). In fact, these are genuine properties of episodic memories (Conway, 2009). On a more pragmatic point, these results also imply that the presence of RIF in any given experiment depends on the specific sequence of the experimental design—the selective practice phase must follow the study phase and no interim retrieval of studied items should take place in order to elicit RIF.

An inhibitory explanation of RIF is at odds with these results at a first glance. It is because this theory assumes that the unpracticed competitors would compete for retrieval during practice and this competition is then resolved by active inhibition, which renders competitors less accessible for later recall (Anderson et al., 1994, 2000; Anderson and Bell, 2001). It is reasonable to assume that unpracticed items would compete for retrieval during practice independently whether these items were retrieved previously or not. Moreover, accepting that initial retrieval strengthened these items, it is plausible to assume that competitors compete even stronger following initial retrieval testing. Therefore, later selective retrieval should induce forgetting on related competitors with and without initial testing of the entire learning set.

However, recently Anderson and Levy (2011) described some fundamental prerequisite for applying inhibition as an explanation for the presence or the lack of RIF in a given experiment. This is the demand/success trade off principle that is proposed to apply inhibitory explanation in a functional theoretical frame for RIF. This principle holds that the relation between interference of competitors and the size of inhibition follows a non-monotonic function (Anderson and Levy, 2011; Detre et al., 2013; see experimental evidence in Keresztes and Racsmány, 2013). That is because inhibition is imperfect and failure of inhibition will influence final accessibility of competitors, therefore RIF reflects the joint influence of inhibition demand and failure rate. An inhibitory theory can explain the lack of RIF following initial retrieval by either assuming that inhibition of competitors failed because of earlier retrieval of these items or by assuming that initial testing of competitors decreased the demand of inhibition. If the failure of inhibition diminished RIF following initial retrieval in our experiments, then we should assume that the same failure of inhibition influenced the success of practice phase too. As it was described by Anderson and Levy (2011) the same inhibitory processes should be active during the practice and the final recall phases of the experiment. Accepting this, we should assume that inhibition failure decreased the success rate of the practice phase and the benefit of practice on final recall of practiced exemplars. This is certainly not the case, both the practice success and the benefit of practice on final recall were the same in conditions with and without initial testing. Therefore failure of inhibition cannot explain the lack of RIF following initial retrieval. The other possibility is that initial retrieval decreased the demand of inhibition during practice, as a consequence there was no need to elicit inhibition on competitors. There is no direct way to test this hypothesis in our experiments, however, there are indirect evidences underlying this assumption in the literature of retrieval-enhanced learning (Roediger and Karpicke, 2006b).

A couple of recent experiments have found the retrieval of cue-target associations decreased the interference of these associations with learning of new associations to the same cues (Szpunar et al., 2008; Halamish and Bjork, 2011). Based on this, it seems plausible to assume the initial testing of the entire learning test significantly decreased the interference between exemplars during practice, and the level of interference between target and competitor items did not trigger inhibition. Although the lack of inhibition demand can be used in explaining the results of the present study by inhibition, inhibitory theories can offer no mechanism to explain why initial retrieval decreased the later demand of inhibition.

Interference theories of RIF are at odds with our results because these theories do not predict that an initial retrieval attempt should modulate the effect of later selective practice. The latest version of interference models (Verde, 2013), assumes that RIF is the result of a sampling failure. In this model, retrieval strengthens the context-item associations, whereas restudy strengthens cue-item associations. Accepting this, we should assume that initial retrieval of the entire learning set strengthens the context-item associations equally for targets and competitors. As a consequence, relative association strengths, which determine the sampling process, remain unaffected; the following selective retrieval practice should still lead to RIF.

Context-based accounts suggest mechanisms inherent to episodic retrieval processes to explain the current pattern of results. Context-based accounts of RIF and retrieval-enhanced learning (Jonker et al., 2013; Karpicke et al., 2014) emphasize the role of context change between initial study of category-member pairs on the one hand, and selective retrieval and final recall on the other. These accounts predict that an initial retrieval of the entire learning set after the study phase will already have participants change their mental context and later selective retrieval practice will cause no further change in this mental context. As a consequence, the context of the initial retrieval will be the active context at final recall.

Altogether context-based accounts of RIF assume that in the retrieval practice paradigm, selective retrieval restricts the search set through encoding a biased contextual information into an episodic memory representation, but an initial, non-selective, retrieval of the entire learning set before the selective retrieval can hinder this search set restriction.

A recent account of the testing effect—the episodic context account of retrieval-enhanced learning (Karpicke et al., 2014)—can be regarded as an extension of episodic and context-based accounts of RIF to a broader range of episodic memory phenomena. This theory aims to explain a range of long-term changes that occur as a consequence of retrieval. Although a detailed presentation of this theory is beyond the scope of the present paper, one relevant suggestion of it is that whenever studying and retrieval take place in different temporal contexts, retrieval will reinstate and update the study context by encoding a composite of study and retrieval contexts (see Karpicke et al., 2014; Lohnas and Kahana, 2014). On a later test participants will use the updated compound context to restrict the search set—the group of items considered as candidates for retrieval (Karpicke et al., 2014). According to this account, the retrieval practice paradigm involves manipulations that produce different kinds of contexts for practiced and unpracticed categories. That is selectively practiced categories will have the compound context of the study and the practice phases, whereas the unpracticed categories will have solely the context of the study phase. Another specificity of the retrieval practice paradigm is that participants typically retrieve practiced items more than once (the most frequently applied procedure involves three retrieval practice cycles). This procedure enables participants to encode strong and detailed contextual information for the practiced sets. As a consequence, they probably will rely more on the context of retrieval practice than on the context of study phase during final recall, and this will bias the recall output in favor of practiced items over unpracticed ones, as unpracticed items have no associations to context features of the practice phase. In contrast, participants will reinstate the context of the study phase whenever they use an unpracticed category label as a retrieval cue.

In other words, according to this account—also in line with the context-based explanations of RIF–RIF is due to a core attribute of retrieval; it is present when the updated context of the selective retrieval allows the participants to restrict their search set mainly for the practiced items. The initial retrieval in our experiments let participants to update the context of the study phase with the context of the initial retrieval. As a consequence, receiving the category cue they could use the compound context of study and initial retrieval while attempting to retrieve unpracticed items from practiced categories at final recall. In this view, retrieval is the key process that enhances long-term accessibility of retrieved memories and it is the process that can hinder retrieval of items through search set restriction or can shield against the adverse effect of later selective retrieval.

### Conflict of Interest Statement

The authors declare that the research was conducted in the absence of any commercial or financial relationships that could be construed as a potential conflict of interest.

## Footnotes

1 Although a few studies with clever designs (e.g., Storm et al., 2006, 2008) did investigate the effect of iterating retrieval practice cycles and relearning cycles after the study phase in retrieval practice paradigms, these studies focused on the effect of adaptive forgetting on later relearning, and were not designed to look at the effect of an initial, non-selective retrieval on the negative effects of later selective retrieval.

2 Since some of our hypotheses concerned finding null-effects (i.e., no RIF), we performed preliminary power calculations with G*Power (Faul et al., 2009) using earlier published (Racsmány et al., 2010) and unpublished data from a retrieval practice paradigm with similar material and population from our lab. To achieve a power of (1 – β) = 0.8 to detect RIF, with a two-sided paired-samples *t*-test, we needed to include 25 participants per condition. Therefore we settled on 30-participant samples for all our experiments—a number common in retrieval-practice paradigms.
